# Microalgal Cultivation in Secondary Effluent: Recent Developments and Future Work

**DOI:** 10.3390/ijms18010079

**Published:** 2017-01-01

**Authors:** Junping Lv, Jia Feng, Qi Liu, Shulian Xie

**Affiliations:** School of Life Science, Shanxi University, Taiyuan 030006, China; lvjunping024@sxu.edu.cn (J.L.); fengj@sxu.edu.cn (J.F.); liuqi@sxu.edu.cn (Q.L.)

**Keywords:** microalgae, the treatment of secondary effluent, pollutants removal, biomass production, lipid accumulation

## Abstract

Eutrophication of water catchments and the greenhouse effect are major challenges in developing the global economy in the near future. Secondary effluents, containing high amounts of nitrogen and phosphorus, need further treatment before being discharged into receiving water bodies. At the same time, new environmentally friendly energy sources need to be developed. Integrating microalgal cultivation for the production of biodiesel feedstock with the treatment of secondary effluent is one way of addressing both issues. This article provides a comprehensive review of the latest progress in microalgal cultivation in secondary effluent to remove pollutants and accumulate lipids. Researchers have discovered that microalgae remove nitrogen and phosphorus effectively from secondary effluent, accumulating biomass and lipids in the process. Immobilization of appropriate microalgae, and establishing a consortium of microalgae and/or bacteria, were both found to be feasible ways to enhance pollutant removal and lipid production. Demonstrations of pilot-scale microalgal cultures in secondary effluent have also taken place. However there is still much work to be done in improving pollutants removal, biomass production, and lipid accumulation in secondary effluent. This includes screening microalgae, constructing the consortium, making use of flue gas and nitrogen, developing technologies related to microalgal harvesting, and using lipid-extracted algal residues (LEA).

## 1. Introduction

Wastewater treatment plants (WWTPs) have played a significant role in improving water environments and the efficient use of water resources. Secondary effluent from the anaerobic–anoxic–oxic (A^2^/O) process (the most commonly used wastewater treatment process in China), or processes derived from this, is considered to be low in organics, nitrogen, and phosphorus [1]. Generally the biological removal of nitrogen via an A^2^/O process is mainly dependent on nitrifying bacteria and denitrifying bacteria [2]. However some features of the process, such as slow growth rate, sensitivity to toxic shocks, pH, and temperature change, are not conducive to the stable removal of nitrogen from wastewaters [3]. In China in particular, many WWTPs are facing pressures to improve effluent quality to meet the Chinese National First A-level Sewage Discharge Standard. This means that secondary effluent should not exceed the threshold of chemical oxygen demand (COD) 50 mg/L, ammonium 5 mg/L, total nitrogen (TN) 15 mg/L, and total phosphorous (TP) 0.5 mg/L. More importantly, some reports show that the threshold of nitrogen and phosphorus causing eutrophication in streams is 0.21–1.2 and 0.01–0.1 mg/L, respectively [4]. Tertiary treatment is, therefore, necessary to reduce the risk of eutrophication. Currently tertiary treatment technologies are developing rapidly, focusing on coagulation–sedimentation, adsorption, ion exchange, membrane technology, biological filtering, ozonation, and biological nutrient removal (BNR) process, etc. [5,6,7,8,9,10]. Specifically facilities have been upgrading their BNR process to meet the new standards for nitrogen and phosphorous discharges. At present, the most effective BNR processes are the Modified Bardenpho and the University of Cape Town (UCT) treatment process, which exhibit excellent nitrogen and phosphorus removal efficiency, as reviewed by Arita et al. [11]. Nevertheless these techniques have many disadvantages; for example, the instability of the treatment effect, high investment, high treatment cost, and difficulty in being popularized in large scale. Thus efficient, stable, low cost tertiary wastewater treatment systems are essential in reducing nitrogen and phosphorus concentrations in secondary effluent.

Fossil fuels are, currently, the main source of energy for human production activities. Excessive consumption of them accelerates the emission and accumulation of CO_2_, which results in the greenhouse effect and aggravates global climate change [12]. The combustion of fossil fuels also contributes to gaseous pollutants, such as SO_2_, NO_x_, CO, ozone, and volatile organic compounds and may have adverse effects on human health and the environment [13]. Biodiesel appears to be an attractive partial alternative to fossil fuels and a way to reduce carbon emissions and reduce the risk of environment pollution from nitrogen oxides and sulfur oxides. At present, biodiesel derived from oil crops, animal fats and waste oil plays an effective part in addressing the problems caused by the use of fossil fuels [14,15,16,17]. These bioresources do, however, have some disadvantages. For example, the production of oil crops requires huge tracts of arable land and fresh water [15], and the production of animal fats may not be sufficient to replace fossil fuels [17]. It is essential that we seek new bioresources for biodiesel production that need only limited or no additional arable land; that need minimal clean water or can use wastewater; and that, simultaneously, have high biomass and lipid productivity.

Microalgae consist of a wide range of autotrophic organisms. They have comparable photosynthetic efficiency to higher plants, rapid growth rate, and notable adaptability. Carbon, nitrogen, and phosphorus are essential elements for microalgal growth and can be effectively used via different metabolic pathways [18]. They therefore have potential advantages for the removal of pollutants from wastewater.

Some microalgae have been shown to be capable of doubling their biomass several times per day. For example, *Ochromonas danica*, grown in a system containing 40 g/L waste cooking oil with acid values of 10.7 mg KOH/g, has a short doubling time of 12.1 h [19]. This short harvesting life will allow multiple and continuous harvesting of biomass throughout the year. Compared to many types of plants, microalgae are easy to cultivate and can produce a high yield of lipid for biodiesel production. As indicated by Nascimento et al. [20], microalgae with a lipid content varying from 13% to 49% have biodiesel productivity ranging from 3.4 to 23.0 m^3^·ha^−1^·year^−1^ in open raceway pond systems. By contrast, the biodiesel productivity of soybeans is only 0.446 m^3^·ha^−1^·year^−1^ [21]. Also, as reviewed by Bohutskyi and Bouwer [22], Harun et al. [23], and Sialve et al. [24], the microalgal biomass remaining after lipid extraction can be used to generate methane. Coupling biodiesel and methane production provides significant energy advantages, along with the sustainability and economic benefits from nutrient recycling. Microalgae can also fix CO_2_ from flue gas to enhance biomass and lipid production [25,26]. This mitigates the greenhouse effect. Microalgae can produce neutral lipids, which are particularly suitable as a potential alternative to fossil fuel. Accordingly, compared to existing bioresources, microalgae are regarded as an excellent alternative for biodiesel production.

## 2. Advances in Microalgal Cultivation in Secondary Effluent

### 2.1. The Feasibility of Microalgal Cultivation in Secondary Effluent

Cai et al. [18], Chen et al. [27], and Zeng et al. [28] reviewed many investigations into lipid accumulation and the biological removal of nitrogen and phosphorus via microalgae in diverse wastewaters. There are some challenges in cultivating microalgae in wastewater, including unbalanced N/P ratio, the presence of bacterial contamination and competitors, low biomass density and lipid content, and incomplete removal of nutrients [29,30,31]. Nevertheless these studies effectively demonstrate that wastewaters can be an excellent medium for biomass production and lipid accumulation and that nitrogen and phosphorus are also removed from wastewaters.

The physical and chemical characteristics of secondary effluent are completely different from those of municipal wastewater, anaerobic digestion effluent, industrial wastewater, and agricultural wastewater. Nearly all pollutants with high biodegradability in wastewaters are degraded in WWTPs, and the biodegradability of the remaining organic compounds in secondary effluent is poor. It has been reported that the ratio of BDOC/DOC (BDOC: biodegradable dissolved organic carbon; DOC: dissolved organic carbon) in secondary effluent is around 0.25 [32]. The lack of a carbon source is, therefore, a serious challenge for microalgal cultivation with secondary effluent. Li et al. [33] isolated a freshwater microalga, *Scenedesmus* sp. (LX1), and cultivated it with secondary effluent to investigate its ability to remove pollutants and accumulate lipids. The results indicated that *Scenedesmus* sp. (LX1) was well adapted to secondary effluent. The microalgal biomass and lipid content was 0.11 g/L and 31%–33%, respectively, after 15 days batch cultivation. At the same time, concentrations of nitrogen and phosphorus in the secondary effluent were decreased by 98% and 98.5%, respectively. Several other microalgae, such as *Botryococcus braunii* and *Chlorella ellipsoidea* (YJ1), have also been grown in secondary effluent for biomass production and pollutant removal, although the microalgal biomass was below 0.5 g/L [34,35]. Importantly the lipid content of *C. ellipsoidea* (YJ1) was up to 43% [26]. In other studies, microalgal lipid accumulation has been strengthened by nutrient starvation [35]. Consequently, although biomass production may be low, high lipid content can be achieved with low nutrient concentrations in secondary effluent.

Some filamentous, mat-forming cyanobacteria affiliated to *Phormidium* have been isolated from Arctic, subarctic, and Antarctic environments to investigate their potential for tertiary wastewater treatment in cool climates. These cyanobacteria exhibited superior growth and high phosphate uptake rates under cold temperatures (10 and 5 °C, respectively) and formed aggregates that could readily be harvested by sedimentation [36,37]. These interesting studies provide avenues for further research and lay the foundations for microalgal cultivation and tertiary wastewater treatment in regions of high altitudes and latitudes with cold climates.

### 2.2. Microalgal Lipid Production in Secondary Effluent

Although low nutrient concentrations in secondary effluent are beneficial to microalgal lipid accumulation, microalgal biomass production is inhibited (as seen in the studies described in Section 2.1), and this shortcoming needs to be addressed. Some studies have found that relatively high biomass was obtained when microalgae were cultivated in secondary effluent. For example, biomass production of *S. obliquus* and *B. braunii*, cultivated in secondary effluent, was up to 1.684 and 1.88 g/L, respectively [38,39]. The possible reason for this was supplementation with CO_2_ as an inorganic carbon source, promoting microalgal growth. Park et al. [40] found that three strains, *Chlamydomonas debaryana* AMB1, *C. sorokiniana* RBD8 and *Micractinium* sp. RB1b, showed large increases in biomass productivity when cultivated mixotrophically in secondary municipal wastewater supplemented with glycerol. This indicates that the exogenous supplement of an organic carbon source helped to strengthen the biomass production of microalgae. The adaption to secondary effluent was species-dependent. Compared to the high biomass production of *Muriellopsis* sp. and *S. subpicatus* in secondary effluent, *C. vulgaris*, *C. fusca*, *Chlorella* sp., and *Pseudokirchneriella subcapitata* had low biomass production in the same conditions [41]. Similar results were also found in other investigations [39]. Selection of the most appropriate microalgae is clearly critical to producing high biomass in secondary effluent.

### 2.3. Microalgal Immobilization

Immobilization technology is a good choice for harvesting or separating microalgal biomass from reactors. However the main focus of microalgal immobilization for secondary effluent treatment has been to enhance removal of pollutants. At present, the most widely used material for microalgal immobilization is alginate. A major advantage of alginate beads containing immobilized microalgae is that immobilized microalgae do not suffer extreme physical and/or chemical changes in the process of immobilization. In addition, the excellent permeability, low or null toxicity, and high transparency of alginate matrix provide a suitable environment for immobilized microalgae. As reported, *Chlorella* sp. and *Scenedesmus* sp., immobilized by alginate matrix, showed high removal efficiency of nitrogen and phosphorus (more than 90%), when some other factors (cell density and starvation) were simultaneously optimized [42,43,44]. Chitosan- and carrageenan- immobilized microalgae (*Phormidium*, *S. bicellularis*, *S. quadricauda*) were also efficient in removing nitrogen and phosphorus from secondary effluent [45,46,47]. Although the removal of pollutants is mainly dependent on microalgal assimilation, immobilized matrixes also promote the removal of pollutants. As discussed by Tam and Wong [48], the presence of calcium ions in alginate matrix, together with elevated wastewater pH, favored the precipitation of phosphate as calcium phosphate. Similarly the calcium dissolved as a result of abrasion of the chitosan particles in wastewater seemed to be conducive in reducing orthophosphate levels in the effluent [46]. Of course, the possible linkages or ionic exchanges between the orthophosphate and chitosan amide groups may provide exchange or fixation sites for orthophosphate [46]. Covarrubias et al. [49] found that the populations of *C. sorokiniana* and *Azospirillum brasilense* in non-sterile secondary effluent were significantly lower than in sterile wastewater when they were cultivated as free suspensions, and the population of wastewater bacteria and natural microfauna increased. However immobilization of *C. sorokiniana* and *A. brasilense* in polymer Ca-alginate beads significantly enhanced their populations in non-sterile secondary effluent. Alginate beads clearly provided a beneficial physical barrier against native microorganisms in secondary effluent.

Attaching to surfaces or matrices in the natural environment is normal for many microorganisms [50], and microalgae can attach to different kinds of materials. *C. vulgaris* and *Scenedesmus* sp. have been found to attach to carriers made from bundles of polypropylene fibers, and the microalgal biofilm photobioreactor thus created significantly reduced more than 90% of ammonium and total phosphorus in secondary effluent [51,52]. Concrete slabs have also been used as carriers for microalgal growth when pollutants from secondary effluent were removed effectively [53]. A rotating algal biofilm reactor (RABR) for wastewater treatment with in situ biomass harvest has been reported in some studies [54,55]. The RABR consists of a cylinder provided with a growth surface partially submerged in wastewater. The cylinder is rotated to alternately expose the growth surface to the wastewater and to air. Of course, effective growth substrata are crucial to microalgal attachment and biofilm formation in RABR [54,55]. Additionally the Algal turf scrubber (ATS), using a natural mixed assemblage of attached periphyton, microalgae, and bacteria, has been developed [56,57]. The ATS is a long inclined flow-way that supports a biofilm of microalgae and bacteria. When wastewater washes over the flow-way in a series of pulses, pollutants are removed effectively. To date, ATSs have been used to provide tertiary treatment of municipal wastewaters and have performed very well [58,59].

Although all the above examples demonstrate that immobilized microalgae were beneficial in improving pollutants removal, partial biological degradation of alginate beads was observed during tertiary wastewater treatment [60]. Immobilization also had some negative effects on microalgal growth rate and biomass productivity [61]. Other unfavorable factors, such as low light penetration and high cost, have limited the commercial application of microalgal immobilization in tertiary wastewater treatment [62].

### 2.4. Construction of a Microalgal Consortium

Microalgae are generally grown as monocultures for harvesting high-value products. However to take advantage of the synergetic growth of microalgae and bacteria, a consortium of the two has been developed to treat wastewater with high levels of organic pollutants [63,64,65]. The consortium is also robust in the face of environmental fluctuations, resists the invasion of other species, and enhances the stability of reaction systems [66]. De-Bashan et al. [67] found that a mixed culture of microalgae (*C. vulgaris* or *C. sorokiniana*) and a bacterium (*A. brasilense* strain Cd) immobilized into small alginate beads improved the efficiency of nitrogen and phosphorus removal from secondary effluent, compared to microalgae alone. The mixed culture removed 100% ammonium, 15% nitrate, and 36% phosphorus, a result that was superior to microalgae alone (75% ammonium, 6% nitrate, and 19% phosphorus). To identify the appropriate combination of microalgae to enhance biomass productivity in secondary effluent, *Scenedesmus* sp. (LX1), *C. ellipsoidea*, and *Haematococcus pluvislis*, were chosen for a study of the growth characteristic of different microalgal combinations in secondary effluent [68]. The intrinsic growth rate of the three microalgae in the mixed culture was higher than when they were grown as monocultures. Biomass productivity in the mixed cultures of *Scenedesmus* sp. (LX1) and *H. pluvialis* increased by 64% and 42%, respectively, compared to monocultures of *Scenedesmus* sp. (LX1) and *H. pluvialis*. These cases suggest that the efficiency of pollutants removal and biomass productivity are strengthened by constructing the consortium.

### 2.5. Pilot-Scale Culture of Microalgae in Secondary Effluent

Pilot-scale culture of microalgae in secondary effluent has been developed gradually. Van Coillie et al. [69] tested the feasibility of a tertiary treatment using *Scenedesmus* sp. at pilot-scale level. An outdoor tank with a capacity of 15,000 L was used for batch culture. Results showed that nutrient removal was 95% for total inorganic nitrogen and 60% for PO_4_^3−^ and that the biomass productivity was 0.39 mg·L^−1^·h^−1^. McGinn et al. [70] operated a 300 L proprietary Brite-Box photobioreactor for microalgal cultivation in secondary effluent. They showed that biomass productivity averaged 130 mg·L^−1^·day^−1^, and nitrogen and phosphorus removal could be up to 90%, when average hydraulic retention times ranged from 6.55 to 6.65 days and from 6.50 to 6.56 days, respectively. Arbib et al. [71] operated a 530 L high rate alga pond (HRAP) and a 380 L airlift tubular photobioreactor (TPBR) to remove nitrogen and phosphorous from WWTP effluent. Maximum areal productivity was 8.26 and 21.76 g suspension solid (SS)·m^−2^·day^−1^ for HRAP and TPBR, respectively; total nitrogen removal averaged 89.68% and 65.12% for TPBR and HRAP, respectively; and, for total phosphorus removal, TPBR and HRAP averaged 86.71% and 58.78%, respectively. There was no significant lipid content difference between the reactors, which was about 20.8%. Based on these studies, it appears that tertiary wastewater treatment and biomass production by microalgal cultivation is feasible at a pilot-scale level, and this provides a basis for large-scale cultivation of microalgae in secondary effluent.

## 3. Future Research into Microalgal Cultivation in Secondary Effluent

The progress in research examined here clearly indicates that microalgae have some potential to biomass production, lipid accumulation and pollutant removal in secondary effluent. However considerable work remains to be done to enhance lipid productivity, wastewater treatment efficiency, and microalgal harvesting in the future.

### 3.1. Screening Microalgae and Constructing the Microalgal Poly-Culture and Microalgal–Bacterial Co-Culture Consortium

Many microalgae, such as *Chlorella*, *Scenedesmus*, and *Botryococcus*, have been screened for biomass production [72,73,74]. Nevertheless, compared to those microalgae growing well in municipal wastewater, the diversity of microalgae growing in secondary effluent is low. The main groups focused on *Chlorella* and *Scenedesmus* (Table 1). In addition, the removal efficiency of nitrogen and phosphorous and lipid productivity during long-term operation was not good (Table 2). It was found to be essential to screen more microalgae to determine their suitability for growth in secondary effluent and to evaluate their lipid accumulation and biomass production potential. Whether oil-rich microalgae growing well in municipal wastewater could adapt to the new environmental conditions of secondary effluent remains to be explored.

The screening of native microalgae has been reported to be a suitable technique for integrated wastewater treatment and biomass accumulation [89]. Bohutskyi et al. [77] reported that the microalgae performing best in unsterilized wastewater were found to be clustered on the same branch of the phylogenetic tree. Combining microalgal screening and molecular phylogeny will be important in identifying the phenotypic traits and genes responsible for superior growth in wastewater. Following the fast development of omic technologies (e.g., genomics, transcriptomics, and proteomics), a large number of genomic, proteomic, and transcriptional data have become available for elucidating gene properties relevant to oil accumulation [90,91,92]. These will be valuable in the genetic engineering of microalgae for lipid production. Nevertheless there have been no reports of combining microalgal screening and omic technologies for microalgal growth and pollutant removal. Revealing the genetic traits of microalgal growth and nutrients removal via omic technologies and applying genetic engineering to improve productivity and nutrients removal abilities will be the focus of future research.

Even though the appropriately screened microalgae have shown excellent growth performance, their scaled cultivation with real secondary effluents in open systems is subject to strong competition (from local microalgal and microbial communities, when single microalgal bioprocesses are considered) and predation [93]. Single microalgal cultivation is also particularly sensitive to sudden changes in environmental conditions (such as light, temperature and nutrient availability) [93]. As reviewed by Fouilland [93], microalgal communities with high species richness and specific metabolic capacities were not only able to capture a high proportion of available resources for growth but also had their resilience enhanced. Microalgal–bacterial co-cultures also helped to ensure successful, intensive, stable microalgal production [93]. At present, preliminary attempts to establish a consortium for biomass production and pollutant removal in secondary effluent are being carried out, as seen in Section 2.4. Both microalgal poly-cultures and microalgal–bacterial co-cultures exhibited better performance than microalgal mono-cultures. However this research was in the early stages of tertiary wastewater treatment; importantly the consortium only contained two microalgae or an artificial combination of microalgae and bacteria. Constructing the consortium by natural selection, or by using an artificial assemblage of robust microalgal poly-cultures and microalgal–bacterial co-cultures with higher species richness and specific metabolic capacities, is of important practical value for tertiary wastewater treatment and lipid production.

### 3.2. Carbon Supplementation by CO_2_ Sequestration in Secondary Effluent

For microalgal cultivation, aeration provided by CO_2_ is not only beneficial to carbon accumulation via photosynthesis, but also contributes to mixing the culture, preventing settlement, and maintaining homogeneous conditions. Currently supplying pure CO_2_ or flue gas for microalgal cultivation prevails, and biomass and lipid content have thereby been increased in many microalgae [94]. Considering the serious lack of a carbon sources in secondary effluent, it has been necessary to investigate the relationship between CO_2_ supplements and biomass and lipid accumulation. Compared to microalgal cultivation without CO_2_ supplement, the biomass production and lipid content were enhanced in most cases when the concentration of pure CO_2_ supplied in secondary effluent ranged from 1% to 5% (Table 1). Flue gas produced by human activities is huge and can be regarded as a rich source of CO_2_. Apart from CO_2_, flue gas contains N_2_, NO_x_, SO_x_, C_x_H_y_, CO, particulate matter, halogen acids, and heavy metals, which are toxic and likely to inhibit the microalgal growth if added to secondary effluent [95]. CO_2_ concentrations in flue gas were also found to be up to 6%–15%, which can inhibit the growth of some microalgae [96]. This phenomenon was also found when microalgae were cultivated in secondary effluent with 15% CO_2_ (Table 1). It is, therefore, critical to optimize the use of CO_2_ from flue gas for biomass production and lipid accumulation of microalgae in secondary effluent.

### 3.3. Improving the Use of Nitrogen in Secondary Effluent

Ammonium has been the preferred form of nitrogen because a redox reaction is not involved in its assimilation by microalgae [18]. Nitrate is used by microalgae only when the ammonium is low or depleted in the wastewater [18]. For WWTPs, the difference in processes or operation parameters resulted in differences in the abundance and richness of nitrifying and denitrifying bacteria [97,98]. These led to different nitrifying and denitrifying activities and then influenced the concentration of ammonium and nitrate in secondary effluent. Seasonal variation was also found likely to influence nitrifying and denitrifying activity via the temperature fluctuation [99]. Thus, to some extent, the ratio of ammonium/nitrate in secondary effluent was not constant and showed dynamic changes (Table 3). Enhancing the efficiency with which different forms of nitrogen (especially nitrate) are removed, in the light of possible nitrogen fluctuations in secondary effluent, will be a major concern.

### 3.4. Growth of Microalgae in Unsterilized Secondary Effluent

It has been difficult to sterilize or filter large amounts of secondary effluent to eliminate the potential effects of native organisms on microalgae in large-scale microalgal cultivation. Secondary effluent commonly contains some microorganisms, viruses, and predatory zooplankton, although their diversity and abundance are lower than in untreated wastewater. Microalgal growth is, therefore, likely to be affected when microalgae are cultivated in unsterilized secondary effluent. Lee et al. [102] found that *Chlorogonium* sp. outgrew other species in non-sterile secondary effluent. Zhang et al. [103] showed that *Scenedesmus* sp. ZTY1 exhibited good adaptability to secondary effluent. Although the growth rates of *Chlorella* sp. under conditions of non-sterilization were lower than under sterilization, non-sterile circumstances were beneficial for accumulating lipids and removing nutrients from secondary effluent [104]. In contrast, Bohutskyi et al. [77] found that most of the miroalgal species they tested were unable to grow efficiently in unsterilized secondary wastewater effluents. Yu et al. [105] showed that the growth of *C. ellipsoidea* was inhibited in unsterilized, domestic, secondary effluent. The potential mechanism was that soluble algal products accumulated in microalgal culture were used as a carbon source by bacteria and promoted bacterial growth. The overgrowth of bacteria then significantly inhibited the activity of microalgae. Based on the above results, there are some challenges for microalgal cultivation in unsterilized secondary effluent.

There has been considerable effort to promote the growth and lipid accumulation of microalgae in unsterilized secondary effluent. Supplementation with centrate from an anaerobic digester was found to be helpful in promoting biomass production in secondary effluent [106,107]. Appropriate doses of microalgal inoculum and correct light intensity also increased final biomass density and productivity in secondary wastewater [108]. The isolation of specific microalgae with excellent resistance to biotic pollution will be an important future goal. A successful example of screening microalgae with good biomass and lipid production in unsterilized wastewater has been reported [109]. Apart from improving the lipid content of microalgae via genetic engineering [110], it may be possible to enhance microalgal resistance to biotic pollution by genetic manipulation. Ultraviolet pretreatment of wastewater was found to increase microalgal growth rate and significantly reduce native bacterial densities [111]. However there has been almost no use of the above control technologies to enhance the growth of microalgae in unsterilized secondary effluent. Considerable work remains to be done.

### 3.5. Development of Microalgal Harvesting Technologies

Harvesting of the microalgal biomass has been a major bottleneck for biodiesel production, largely because of the small size of some cells (typically in the range of 3–30 µm); the relatively low cell density, especially in the raceways (<0.5 kg/m^3^ of dry biomass); and the large volume of water being harvested [112]. The recovery of microalgae generally requires one or more solid–liquid separation steps. Currently microalgal harvesting technologies include chemical flocculation, centrifugation, gravity sedimentation, filtration and screening, flotation, electrophoresis, and immobilization [113,114]. The immobilization technique, in particular, has been used effectively to separate microalgal cells from secondary effluent. However the huge consumption of chemicals and energy were major challenges. These drawbacks limited the use of these technologies for large-scale microalgal harvesting as they accounted for 20%–40% of the total costs of lipid production [112]. Some chemicals also showed a certain degree of biomass toxicity [115], and the high density of the immobilization matrix was likely to reduce the light penetrating through the reactor, thus affecting metabolic activity [62]. Theoretically self-settlement induced by exogenous selection pressures was beneficial to biomass–water separation. A typical case was the development of aerobic granular sludge by regulating some parameters such as settling time and the mixed liquor volume exchange ratio [116,117]. Valigore et al. [118] regulated hydraulic retention times and solids retention times of laboratory sequencing batch reactors to cultivate settleable microbial (microalgal–bacterial) biomass grown on primary treated wastewater as a biodiesel feedstock. They showed that biomass settleability was typically 70%–95%, and the microbes, aggregated into compact flocs as cultures, aged up to 4 months. Related investigations, however, were only just beginning. In the future, it will be interesting to consider strengthening microalgal–water separation in secondary effluent, screening microalgae of high settleability via exogenous selection pressures.

### 3.6. Use of Lipid-Extracted Algal Residues

A scaled biodiesel production process will generate enormous amounts of lipid-extracted algal residues (LEA), containing major parts of the energy and all nutrients captured by microalgae. Rational use of LEA will help to reduce costs and develop a sustainable microalgal cultivation and biodiesel production process. Anaerobic digestion (AD) is a good choice, as this can convert LEA into a biogas, mainly consisting of CH_4_ and CO_2_, with traces of other gases such as H_2_S [119]. Passing the biogas into the microalgae culture will not only benefit microalgal growth and nutrient removal, but also the purification of the biogas [120,121]. The biogas may be burned to produce electricity and to generate an onsite source for CO_2_ to supplement microalgae [122,123]. AD effluent, which contains many nutrients, can also be partially used as a chemical fertilizer during microalgal growth [106,107]. As elucidated by Bohutskyi et al. [107], the methane production from LEA increased the energy yield from microalgal biomass by more than 30%. Additionally the supplementation of AD effluent during microalgae culture can reduce fertilizer costs by 45%. Coupling biodiesel production and LEA use provides significant energy advantages as well as sustainability and economic benefits from nutrient recycling. However some factors will have a significant impact on the methane yield and productivity, notably (1) the difficulty in biodegrading microalgal cell walls; (2) the high protein content of the microalgal biomass, which results in high ammonium release; and (3) the high sodium concentration in microalgal cells, which may alter the anaerobic process. It will, therefore, be important to optimize LEA anaerobic digestion to ensure the absence of solvent residues inhibiting methanogens and to enhance LEA biodegradability for maximum methane yield, since some LEA fraction may be recalcitrant, as reviewed by some literatures [24,119].

## 4. Conclusions

Sustainability is a key principle in microalgal-based wastewater treatment and biodiesel production. It is important to consider how to minimize the environmental impact and strengthen the economic and social benefits of the process. This review underlines the viability of using secondary effluent as a potential medium for simultaneous microalgal growth and pollutant removal. As elucidated in the review, pollutant from secondary effluent can be effectively removed, and the lipid, as a renewable energy resource, is also accumulated by microalgae via fixing CO_2_. Immobilization of microalgae has significant advantages in promoting pollutants removal and also provides a beneficial physical barrier against native microorganisms in secondary effluent. A consortium of microalgae and/or bacteria results in greater biomass and lipid productivity and more effective removal of pollutants than is found in a mono-culture. Obviously it is feasible to integrate microalgal cultivation, as biodiesel production feedstock, with tertiary wastewater treatment. It is beneficial to reduce the risk of the eutrophication and carbon emissions. In addition, compared to oil crops for biodiesel production, it doesn’t involve the conversion of agricultural land for biodiesel production or affect food security. Therefore it exhibits huge environmental and social benefits. However the high economic cost and the low efficiency (mainly in microalgal growth, lipid accumulation, and pollutant removal) have become the bottleneck of microalgal cultivation in secondary effluent. As potential solutions to these problems, considerable effort will be needed in the future to screen microalgae, construct the consortium, improve the use of flue gas and nitrogen, develop technologies for harvesting microalgae, and use LEA. Coupling biodiesel production and LEA use especially provides significant energy advantages, along with sustainability and economic benefits from nutrient recycling. In general, all these investigations represent potential approaches to integrating tertiary wastewater treatment and microalgal lipid accumulation for biodiesel production.

## Figures and Tables

**Table 1:** Biomass production, lipid productivity, and pollutant removal of microalgae in secondary effluent under batch culture.

Microalgae Species	Free Cell (F) or Immobilization (I)	Preliminary Treatment of Wastewater	The Volume of the Cultivation (L)	Cultivation Time (h)	CO_2_ (%)	Biomass Energy				Nutrients Removal Efficiency (%)					References
						Biomass Production (g·L^−1^)	Biomass Productivity (mg·L^−1^·day^−1^)	Lipid Productivity (mg·L^−1^·day^−1^)	Lipid Content (%)	COD	TN	NH_4_^+^	NO_3_^−^	TP	
a natural algal bloom	F	no treatment	2	240	5	1.884	200.4	-	26.82	-	79	-	-	>98	[38]
*Botryococcus braunii*	F	filtration (0.2 µm) and autoclaving	3	240	1	0.35	-	-	-	-	-	-	>99	>99	[34]
*Botryococcus braunii*	F	filtration	0.5	1000	-	-	288–345.6	-	17.85	-	-	-	-	-	[75]
*Botryococcus braunii*	F	no treatment	9	336	5	1.88	-	-	36.14	-	-	-	79.63	100	[39]
*Chlorella ellipsoidea* YJ1	F	filtration (0.45 µm) and autoclaving	0.3	528	-	0.425	-	12.7	43	-	>99	-	-	>90	[35]
*Chlorella kessleri*	F	no treatment	2	240	5	1.172	132.3	-	20.55	-	>90	-	-	>98	[38]
*Chlorella sorokiniana*	F	autoclaving	1	96	12	0.25	62.5	8	32	-	-	-	100	0	[76]
*Chlorella sorokiniana*	F	no treatment	0.45	240	-	0.1	-	-	-	-	80	-	-	40	[77]
*Chlorella* sp. 227	F	filtration (0.45 µm) or UV-radiation	0.5	216	-	0.41–0.67	-	6.9–22.9	15–31	13.8–24.8	75–92	-	-	84–86	[78]
*Chlorella vulgaris*	F	no treatment	2	120	-	0.76–0.82	73.88–79.82	-	-	-	-	-	-	92	[79]
*Chlorella vulgaris*	F	filtration (0.2 µm)	0.2	168	15	0.29	-	-	30	-	>99	-	-	>99	[80]
*Chlorella vulgaris*	F	no treatment	2	240	5	1.303	116	-	22.02	-	>90	-	-	>98	[38]
*Chlorella vulgaris*	F	filtration	2	168	air ^a^	1.03	171.33	43.52	27.6	-	-	-	94	-	[81]
*Desmodesmus communis*	F	filtration	1	360	2	0.79	23	-	9.3				100		[82]
*Neochloris oleoabundans*	F	filtration (1.2 µm) and autoclaving	0.4	240	5	2.1	233.3	-	-	-	-	>90	78–99	100	[83]
*Ourococcus multisporus*	F	filtration (0.2 µm)	0.2	168	15	0.31	-	-	31	-	>99	-	-	>99	[80]
*Scenedesmus obliquus*	F	filtration and autoclaving	1	192	-	-	-	-	31.4	-	-	>90	-	>90	[84]
*Scenedesmus obliquus*	F	filtration (0.2 µm)	0.2	168	15	0.31	-	-	27	-	>99	-	-	>99	[80]
*Scenedesmus obliquus*	F	no treatment	2	240	5	1.684	201.4	-	19.38	-	>90	-	-	>98	[38]
*Scenedesmus* sp. AMDD	F	filtration (0.2 µm)	0.15	288	unknown concentration	0.13	127.22–132.73	-	11.72–12.08	-	-	>90	-	>90	[40]
*Scenedesmus* sp. LX1	F	filtration (0.45 µm) and autoclaving	0.2	360	-	0.11	-	35	31–33	-	98.5	-	-	98	[33]
*Scenedesmus* sp. LX1	F	autoclaving	0.2	336	5	0.77	-	-	35	-	-	-	-	-	[85]
*Chlorella* sp.	I	filtration and autoclaving	0.35	8	air	-	-	-	-	-	-	100	-	100	[44]
*Phormidium* sp.	I	no treatment	0.5	24	-	-	-	-	-	-	-	>90	>90	>90	[46]
*Scenedesmus bicellularis*	I	unknown	unknown	2	-	1.57–1.86	-	-	-	-	-	100	-	88–100	[42]
*Scenedesmus bicellularis*	I	autoclaving	2.5	2	750–1500 ^b^	-	-	-	-	-	-	42.1–100	-	19.1–99.1	[86]
*Seenedesmus quadricauda*	I	roughly screened	1	3	-	-	-	-	-	-	-	85–100	-	-	[45]
*Scenedesmus* sp.	I	filtration and autoclaving	0.35	4	air	-	-	-	-	-	-	100	-	100	[43]
*Scenedesmus* sp.	I	no treatment	96	72	-	-	-	-	-	0	47.86	96	-	>90	[52]

^a^ Except air, the system was supplied by NaHCO_3_; ^b^ ppm.

**Table 2:** Biomass production, lipid productivity, and pollutant removal of microalgae in secondary effluent under continuous culture.

Microalgae Species	Free cell (F) or Immobilization (I)	Cultivation Time (d)	Hydraulic Retention Time (d)	Biomass Energy				Nutrients Removal Efficiency (%)					References
				Biomass Production (g·L^−1^)	Biomass Productivity (mg·L^−1^·day^−1^)	Lipid Productivity (mg·L^−1^·day^−1^)	Lipid Content (%)	COD	TN	NH_4_^+^	NO_3_^−^	TP	
*Chlorella vulgaris*	F	240	0.04–2	0.69–1.289 ^a^	47.5–131.7	-	-	-	54–95.3	-	-	84.4–94.9	[87]
*Scenedesmus* sp. AMDD	F	24	1.48	0.312–0.356	234–267	11.91–15.19	5.14–5.70	-	-	100	-	100	[70]
*Scenedesmus obliquus*	F	112	5	-	21.76 ^b^	-	20.8	-	89.68	-	-	86.71	[71]
*Scenedesmus obliquus*	F	104	10	-	8.26 ^b^	-	20.8	-	58.78	-	-	58.78	[71]
*Scenedesmus* sp.	F	-	5.2	0.5	20 ^b^	-	-	-	-	-	-	-	[88]
*Scenedesmus* sp.	I	91	2	0.1–0.3	-	-	-	21–48.36	36	24–55	-	40–80	[52]

^a^ mg·COD/L; ^b^ g·m^−2^·day^−1^.

**Table 3:** Characteristics of secondary effluent from different sources.

The Number of Secondary Effluent	COD (mg/L)	TOC (mg/L)	BOD (mg/L)	TN (mg/L)	NH_4_^+^-N (mg/L)	NO_3_^−^-N (mg/L)	TP (mg/L)	NO_3_^−^-N/NH_4_^+^-N	References
1	-	7.4	-	6.3	<0.01	4.48	0.39	>448	[34]
2	-	5.5	-	8.9	0.17	7.67	0.04	45.11	[34]
3	45–60	-	-	12.5–23.8	3.8–7.6	-	0.82–1.67	-	[52]
4	24	-	-	15.5	2.5	-	0.5	-	[33]
5	-	-	-	-	0.24	4.94	<0.01	20.58	[82]
6	56	-	-	22.13	4.10	15.12	-	3.69	[100]
7	-	-	-	20.0	7.6	10.3	1.95	1.36	[101]
8	24	-	-	7.0	0.50	-	0.46	-	[35]
9	22.1	-	-	15.5	2.5	-	0.05	-	[35]
10	24.5	-	-	16.7	3.7	-	0.08	-	[35]
11	49.7	-	-	11.9	15.0	0.9	11.5	0.06	[75]
12	-	-	10–19	-	21.62–28.85	-	2.22–3.51	-	[70]
13	-	8.1	-	8.7	9.4	8.5	1.71	0.90	[80]
14	100	-	-	-	21	1.6	5.6	0.08	[38]

TOC: Total organic carbon; BOD: Biochemical oxygen demand.

## Abbreviations

A^2^/O	Anaerobic–anoxic–oxic
AD	Anaerobic digestion
ATS	Algal turf scrubber
BDOC	Biodegradable dissolved organic carbon
BNR	Biological nutrient removal
BOD	Biochemical oxygen demand
COD	Chemical oxygen demand
DOC	Dissolved organic carbon
HRAP	High rate alga pond
LEA	Lipid-extracted algal residues
RABR	Rotating algal biofilm reactor
SS	Suspension solid
TN	Total nitrogen
TOC	Total organic carbon
TP	Total phosphorous
TPBR	Tubular photobioreactor
UCT	University of Cape Town
WWTPs	Wastewater treatment plants
